# Computed tomography angiography scoring systems and the role of skull defects in the confirmation of brain death

**DOI:** 10.1038/s41598-021-94763-8

**Published:** 2021-07-23

**Authors:** Petros Zampakis, Vasilios Panagiotopoulos, Christina Kalogeropoulou, Maria Karachaliou, Diamanto Aretha, Nektarios Sioulas, Sofia Dimoulia, Dimitrios Karnabatidis, Fotini Fligou

**Affiliations:** 1 Department of Radiology, University Hospital of Patras GR, 265 04 Patras, Greece; 2 Department of Neurosurgery, University Hospital of Patras GR, 265 04 Patras, Greece; 3 Department of Anesthesiology and Intensive Care Medicine, University Hospital of Patras. GR, 265 04 Patras, Greece

**Keywords:** Imaging, X-ray tomography

## Abstract

To assess and compare all current computed tomography angiography (CTA) scoring systems for the diagnostic workup of brain death (BD) to digital subtraction angiography (DSA) and clinical tests. Fifty-two patients with a clinical suspicion of BD underwent CTA and subsequently DSA. The diagnostic performance of all current CTA scoring systems was compared to that of DSA, in all patients with a suspicion of BD. A comparison to clinical tests was made only in DSA-positive for BD patients (n = 49), since in DSA-negative BD patients (n = 3) clinical tests were not performed. Further subgroup analysis was performed in relation to skull defects (SDs) stratification. Statistical analysis was conducted by applying statistics-contingency tables, Cochran’s-Q test and McNemar’s test. The CTA -10, and -7- and all 4-point scoring systems, showed overall sensitivities of 81,6%, 87.8% and 95.9% respectively and 100% specificity, when compared to DSA. In patients with a clinical verification of BD, the CTA -10 and -7-point scoring systems were significantly inferior to clinical tests (p = 0.004 and p = 0.031), while the 4-point scoring systems showed no such difference (p = 0.5). All 4-point scoring systems showed 100% sensitivity in patients with a minor SD or no SD. In patients with a major SD, all CTA scoring systems (− 10, − 7- and all 4-point) were less sensitive (62.5%, 62.5% and 75% respectively). The presence of a major SD was associated with an 8 × relative risk for false negative results in all 4-point scoring systems. CTA showed excellent diagnostic performance in patients with a suspicion of BD. The 4-point CTA scoring systems are the most sensitive for the diagnosis of BD, although in patients with a major SD patient, the role of CTA is ambiguous.

## Introduction

The concept of brain death (BD) has been around for several decades, especially when it became clear that it is not synonymous with cardio-respiratory arrest^1^. The declaration of a dead person unavoidably raises moral, ethical, religious, and legal issues.

Very recently a consensus document, the so-called “World Brain Death Project”, was released for the determination of BD/death by Neurologic Criteria (DNC). Due to these guidelines and in agreement with pre-existing definitions, BD/DNC is defined as “the complete and permanent loss of brain function as defined by an unresponsive coma with loss of capacity for consciousness, brainstem reflexes, and the ability to breathe independently”^2,3^.

According to the most recent guidelines, there are certain clinical requirements for the diagnosis of BD/DNC, mainly including the presence of coma and brainstem areflexia, while the apnoea test is of paramount importance^3^.

An early and prompt diagnosis of BD can be of critical importance, mainly when organ donation is possible.

Unfortunately, clinical tests and such thorough examinations for BD confirmation, are not always possible. There are several clinical scenarios in the real world, where clinical tests, cannot be performed/completed or are suboptimal. For this reason, the concept of ancillary tests was conceived. In general, these ancillary tests are imaging studies that can verify the loss of intracranial blood flow, capable of adequately perfusing brain parenchyma. The same applies for a lesion in a region that equates “whole brain death” with BD/DNC (e.g., an isolated brainstem lesion or a posterior circulation vascular lesion), even if the clinical examination and apnoea test are suggestive of BD/DNC. In this case, BD/DNC should not be diagnosed until supratentorial and infratentorial flow loss is verified^3^.

In many countries these tests are often used in combination with the clinical tests^4,5^, while DSA with 100% sensitivity and specificity, has been considered the “gold standard” imaging technique to diagnose BD, due to^3,6–9^. Other ancillary tests, as summarized in the recent “World Brain Death Project”^3^ include: radionuclide angiography (sensitivity 98%), radionuclide perfusion scintigraphy-SPECT (sensitivity 88.4%), transcranial Doppler ultrasound (sensitivity 90%), magnetic resonance angiography (sensitivity 93–100%) and (CTA) + /− CT Perfusion (sensitivity 52–97% ).

Over the last 20 years, Computed Tomography Angiography (CTA) has been applied as an evolving non-invasive technique, to detect intracranial circulatory arrest, although immense variations in its sensitivity have been reported^10–17^. It is well established that the sensitivity of CTA increases as the number of points in different scoring systems decreases. CTA results are suboptimal in patients with skull defects (craniotomy, craniectomy, fractures), although a new modified score has been suggested to overcome this disadvantage^18^.

To date, all but one of the studies^19^ regarding CTA and BD confirmation were performed after the clinical tests. This approach has some disadvantages, regarding organ donation (delays in system reflexes, psychological preparation of the relatives etc.). In our country, according to the 2015 ACCORD study final report, approximately 60% of BD patients are diagnosed in more than 7 days, following a severe brain injury^20^.

The purposes of our study were: (a) to evaluate and compare the diagnostic performance of all current CTA scoring systems in clinically suspected BD patients, with the “gold-standard”(i.e., DSA), (b) to evaluate and compare the diagnostic performance of all current CTA scoring systems for BD confirmation, with the clinical tests performed after the angiographic studies and (c) to compare all CTA scoring systems for BD confirmation in different subgroups of patients SDs.

## Materials and methods

This single-centre retrospective analysis of routinely collected data was performed in our tertiary hospital that accepts patients from a region with a population reaching one million. All patients were managed in our intensive care unit. This study included patients with a clinical suspicion of BD over a period of 4 years and was approved by the Institutional Ethical Committee of the University Hospital of Patras-Greece, [Research Ethics Board (REB)-study number: 801]. For this type of study, the requirement for informed consent was waived by the same committee [Full name: University Hospital of Patras, Research Ethics Board (REB)-study number: 801]. We confirm that all methods were carried out in accordance with relevant guidelines and regulations.

Due to our national legislation, the confirmation of BD depends on clinical tests only. However, in our hospital which also includes a kidney transplant centre; we routinely use ancillary tests (DSA ± CTA) as an adjunctive method in the workup of clinically suspected BD patients, prior to clinical tests, according to the clinical protocol of our Department of Anesthesiology and Intensive Care Medicine.

Our study participants included a consecutive series of patients evaluated for eligibility at the study location and satisfying certain inclusion criteria.

The inclusion criteria for this retrospective study were as follows:

- Clinical suspicion of BD, defined as the presence of (a) mydriasis > 4 mm, (b) apnoeic coma (patient depended on mechanical ventilation) and (c) non-reversible cause of coma (exclusion of hypothermia, metabolic or electrolytic abnormalities, high levels of sedatives and /or narcotic drugs etc.); and
- Both CTA and DSA were performed on our study patients.

Therefore, over the last 4 years, a total of 67 consecutive patients with a clinical suspicion of BD were recorded, but only 52 underwent both CTA and DSA for BD workup and were therefore included in this study.

Finally, our study population consisted of 28 men (54%) and 24 women (46%) with a mean age of 47 years (SD =  ± 15). The median patient age was 52 years (the minimum and maximum patient ages were 16 and 71 years respectively). The initial causes of coma were ischaemic or non-traumatic haemorrhagic stroke (n = 33, 63%), traumatic brain injury (n = 15, 29%), an intracranial tumour (n = 2, 4%) and infectious disorders (n = 2, 4%).

In our patient cohort, we classified SDs as follows:

No = intact skull, or small hairline fractures without any obvious distension or evidence of compaction;

Minor = SDs due to craniotomies, skull drills or distended fractures with a similar defect size to that of skull drill or craniotomy; and

Major = SDs due to craniectomies or large depressed/distended fractures with a defect size larger than that of craniotomy or a skull drill.

Intracranial pressure was monitored only in our trauma patients without severe coagulation and/or platelet disorders (n = 13, 25%). Evaluation of brain oedema in the remaining patients was performed via a clinical examination and CT scans.

According to our clinical protocol, all our patients underwent the same standardized procedures regarding mechanical support and ancillary tests implementation.

Therefore, following the clinical suspicion of BD, all the subjects underwent cerebral CTA, immediately followed by DSA. The time delay between CTA and DSA was approximately 20–60 min. During all examinations, patients were mechanically ventilated using pressure or volume control mode with the tidal volume adjusted between 6–8 ml/kg, based on an ideal body weight, while the respiratory rate was adjusted to maintain the end-tidal CO_2_ between 4–4.7 kPa, and the mean arterial blood pressure was maintained above 65 mmHg. All patients were normotensive during ancillary tests, while renal function was normal. The time between the clinical suspicion of BD and angiographic tests was 8 to 24 h (depending on the availability).

**Figure 1:**
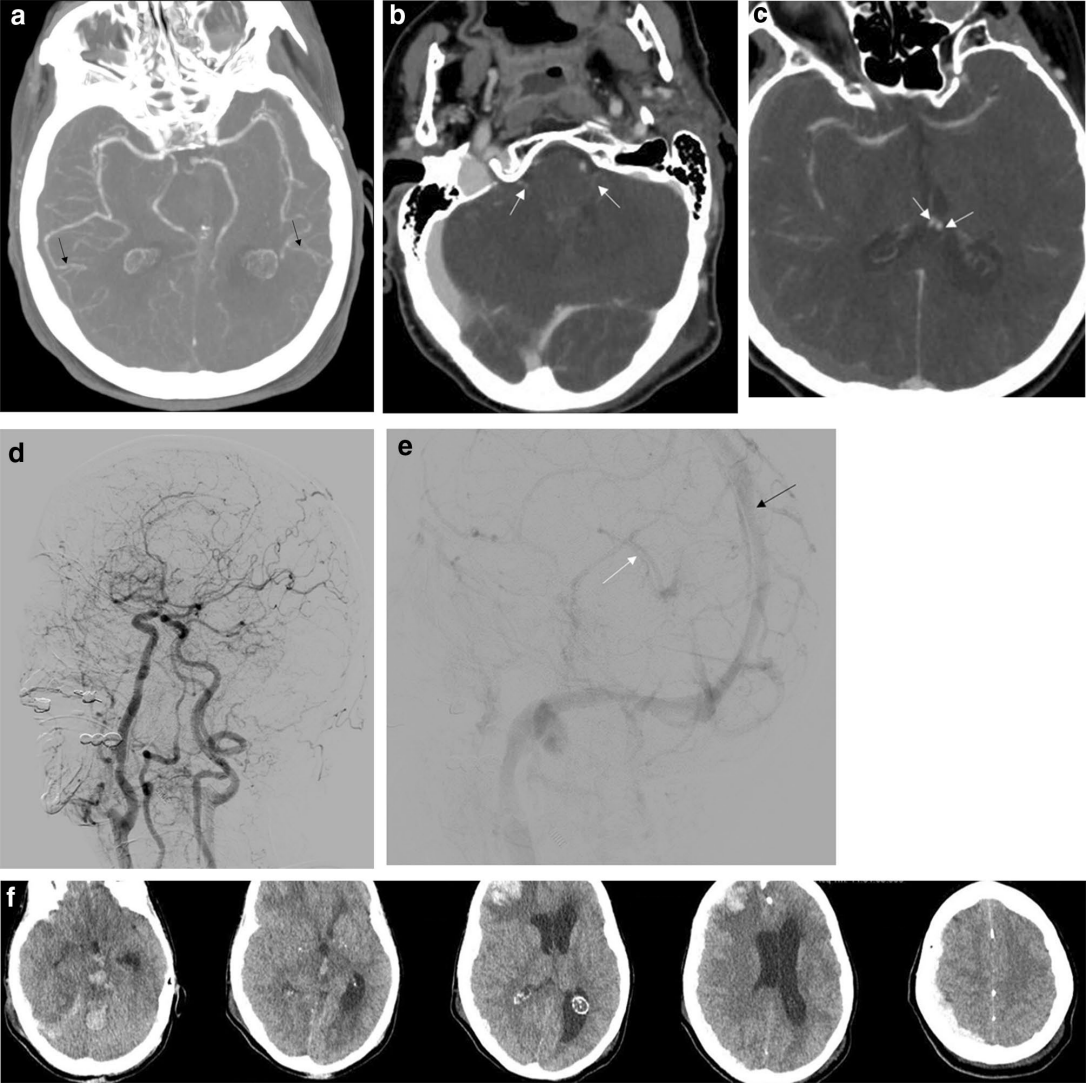
(**a**) Axial CTA-MPR (arterial phase) reveals good patency to all arterial intracranial vessels (including M4 segments-black arrows). (**b**) Axial CTA at the same patient (60 s phase), shows opacification of both superior petrosal veins (white arrows). (**c**) Axial CTA at the same patient (60 s phase), shows opacification of both internal cerebral veins (white arrows). (**d**) Digital Subtraction Angiography at the same patient in arterial phase (LAO oblique view), reveals good flow in all proximal and distal intracranial arteries of the anterior and posterior circulation. (**e**) Digital Subtraction Angiography at the same patient in venous phase (LAO oblique view), shows that superficial (black arrow) and deep venous system (white arrow) is patent. (**f**) Axial plain brain CT at different levels, shows adequate differentiation of gray-white matter and no radiologic signs of brain death, 15 days following the initial angiographic check of the same patient.

In all patients who resulted negative DSA results, for ethical reasons clinical tests were not performed and patients were supported and followed up (n = 3, 6%) (Fig. 1a–f). Major SDs were evident in one of these patients.

In all patients with an angiographic confirmation (DSA) of BD, complete clinical testing for the determination of BD was planned as soon as possible, with appropriate cessation sedation and measurements of depressant and narcotic drug levels when needed (n = 49, 94%). The checklist for the declaration of BD was completed 48 h after ancillary imaging tests (CTA-DSA). Of these 49 patients, major SDs were present in 8 (16.3%), and SDs were present in 8 (16.3%). 33 (67.3%) had no SDs.

As indicated by our national legislation, BD was declared when persistent (> 24 h), non-reversible coma was present and when brainstem areflexia and a positive apnoea test were confirmed. The results of the clinical examination for the determination of BD were recorded in a special “checklist for declaration of brain death” used in our institution. Clinical tests were performed twice with an interval of more than 8 h by a commission of three specialists, including at least one anaesthesiologist and one neurologist or neurosurgeon.

### CTA technique

Cerebral CTA from the level of C2 through the top of the scull was performed in an 80-row detector-dual source scanner (Toshiba Aquillion Prime 80 slices, Toshiba Corp, Japan). A reference non-contrast scan was preceded. The technical parameters of CTA were as follows: images were acquired with a section thickness of 0.5 mm; and scanning parameters were 120 kV, 196 effmAs, and field of view (FOV) 220-mm. In our protocol, 80 ml of iodine contrast agent was injected at a flow rate of 4 ml/s. Following contrast administration, the arterial phase was performed, using a bolus tracking technique (reference standard the cervical external carotid branches), followed by a delayed venous phase at 60 secs. Adequate opacification of the cervical external carotid artery branches was used to confirm that the contrast was injected correctly, and no technical failure was present. Raw data were processed at a dedicated workstation (Vitrea, Medical Image Processing Software, Version 6.0, Vital Images, Inc). All CTA examinations were evaluated by 2 radiologists (> 20 years of experience), a neuroradiologist and a radiologist, who ultimately reached a consensus.

### CTA criteria for BD

Blood flow in all intracranial vessels, including distal intracerebral arteries, as well as the superficial and deep cerebral venous systems was evaluated. For the diagnosis of BD, we used the 10-point scoring system (the so-called CTA-10), the 7-point scoring system (the so-called CTA-7), the 4-point scale introduced by Frampas et al.^12^ (the so-called CTA-F), the revised 4-point scale suggested by Nunes et al.^18^ (the so-called CTA-MF) and the revised venous 4-point scale, proposed by Marchand et al.^16^ (the so-called CTA-M) (Table 1).

**Table 1:** Different CTA scoring systems for determination of brain death.

Vessel	10-point system	7-point system	4-point system (by Frampas)	4-point system (by Marchand)	4-point system (Modified Frampas)
MCA – M4	2	2	2		2*
ACA – A3	2	2			
PCA – P2	2				
BA	1				
ICV	2	2	2		2**
GCV	1	1		2	
SPV				2	
Total	10	7	4	4	4

*MCA* middle cerebral artery, *ACA* anterior cerebral artery, *PCA* posterior cerebral artery, *BA* basilar artery, *ICV* internal cerebral vein, *GCV* great cerebral vein, *SPV* superior petrosal vein.

*Only in arterial phase.

**Only in venous phase.

**Figure 2:**
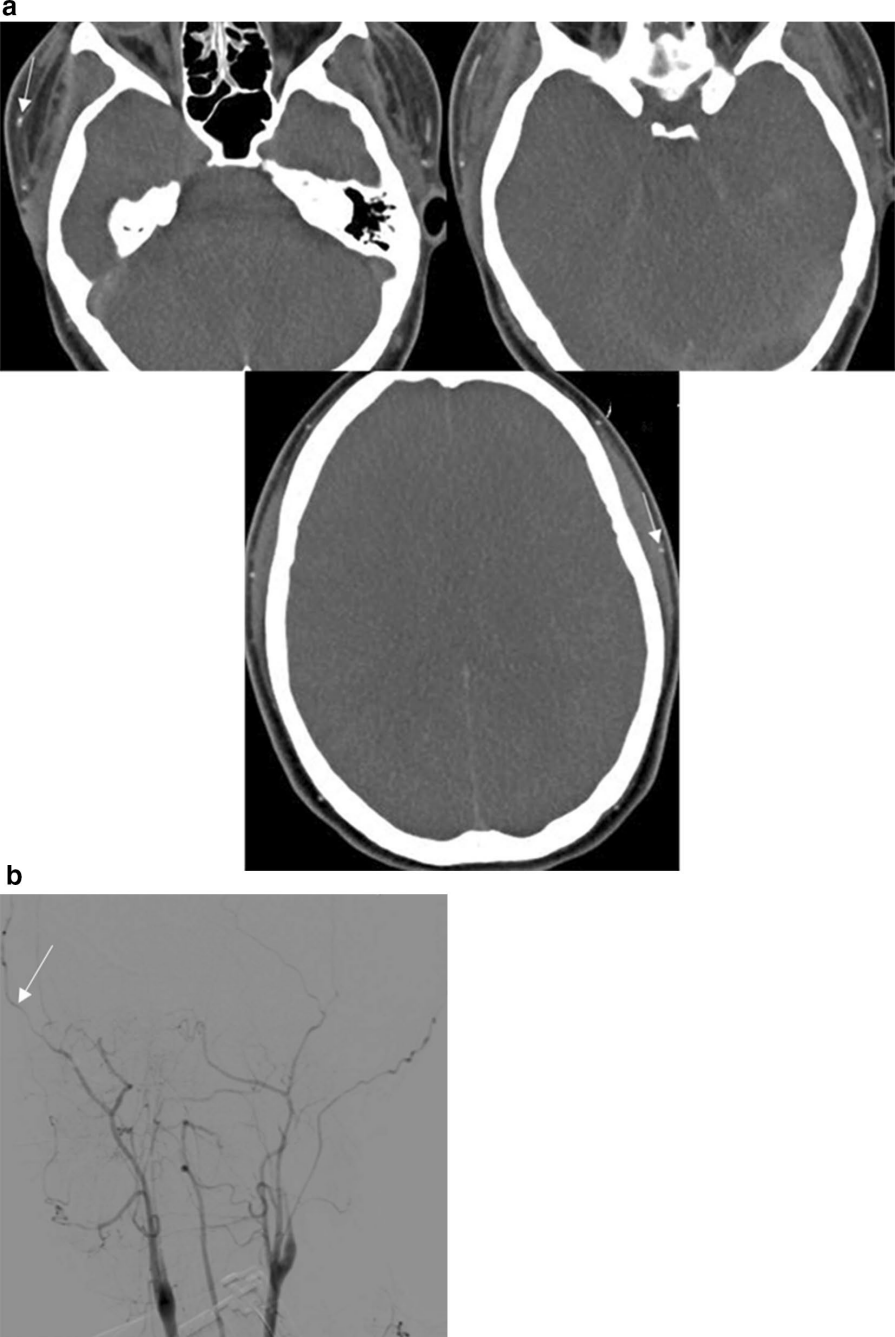
(**a**) Axial CTA (60 s phase) at different levels, shows no intracranial arterial flow or venous flow. Extracranial arterial feeders are visible (white arrows). (**b**) Digital Subtraction Angiography in arterial phase (AP view), shows no intracranial flow, while extracranial arteries are patent (white arrow).

As previously described^15^, a positive result on the 10-point scale (score = 10) confirming the diagnosis of BD, was recorded when the following vessels were not opacified: the bilateral PCA-P2, the basilar artery (BA), the bilateral ACA-A3, the bilateral MCA-M4, the bilateral internal cerebral veins (ICVs), and the vein of Galen (GCV). Scores from 0 to 9 were classified as negative results (excluding the diagnosis of BD) (Fig. 2a).

On the 7-point scale, a positive result (score = 7) was recorded based on the lack of opacification of the bilateral ACA-A3, the bilateral MCA-M4, the bilateral ICVs, and the GCV. Scores from 0 to 6 were classified as negative results for BD.

Using Frampas’s score, CTA was deemed positive for BD when no flow in either the arterial or venous phase, was detected in the M4 segments of the middle cerebral artery, and in the ICVs. According to this score, stasis filling (SF) was defined as opacification of one or more of the following arterial segments (on either the arterial or delayed venous phase): anterior cerebral artery (ACA–A1 and A2), middle cerebral artery (MCA–M1, M2, and M3), and posterior cerebral artery (PCA–P1) without opacification of the cortical M4 arterial branches or deep veins. SF did not preclude BD.

According to the modified-Frampas score, CTA was deemed positive for brain death when no flow was detected in the M4 segments of the MCA in the arterial phase and in ICVs in the venous phase.

Using Marchand’s score, CTA was deemed positive for brain death when no flow in the venous phase was detected in in the superior petrosal veins (SPVs) and in the ICVs. Again, SF did not preclude BD.

### Digital subtraction cerebral angiography

DSA was performed 20–60 min following the CTA with the Allura XPER FD20 Single Plane Angio Lab (Philips Medical Systems-The Netherlands). A typical femoral approach was used in all cases. After positioning a 4–5 F pigtail catheter in the aortic arch, 20 ml of contrast agent was injected at a flow rate of 10 ml/s. We scanned the head and neck by recording a 50-s series with a frequency of 2f/s and using a digital subtraction technique. An experienced interventional radiologist performed all angiograms. Two interventionists, with many years of experience in cerebral angiographies, evaluated the DSA results and ultimately reached a consensus.

### Angiographic criteria for BD

As previously described^15^, DSA was deemed positive for BD, if either of the following criteria were met: Non-filling of intracranial vessels with normal flow in the external carotid arteries, stasis filling—delayed, weak, and persistent opacification of the proximal cerebral arterial segments without opacification of the cortical branches or venous outflow (Fig. 2b).

### Statistical analysis

Contingency tables (frequencies/crosstabs) with the sensitivity, specificity, positive predictive values (PPVs) and negative predictive values (NPVs) and false negative results of five different CTA scoring systems were generated to compare to the results to those of the “gold standard” (i.e., DSA) for the workup of suspected BD patients, and receiver-operating characteristic (ROC) curves were drown. Only the sensitivity and PPV were calculated when CTA results were compared to clinical tests results, since only in patients whose DSA results were positive for BD were clinical tests also performed, and the clinical test results were positive for BD in all these patients.

Cochran’s Q test was used to compare the sensitivities and the proportions of false negative results of a BD diagnosis among ancillary tests (DSA and CTA scoring systems including all 52 patients, and clinical tests and CTA scoring systems including only the 49 patients with positive clinical tests for BD), while it was also used to compare CTA and clinical tests according to patients’ SDs (49 patients).

The significance of differences between tests was assessed by McNemar’s test. More specifically differences between CTA and DSA (52 patients) and differences between CTA and clinical tests (49 patients) were measured.

A multivariate logistic regression model (backward stepwise conditional method) that incorporated sex, age and the presence of SDs (major, minor or no defects) was used to identify independent predictors of false negative CTA results for the different CTA scores compared to DSA (52 patients). P < 0.05 was considered statistically significant.

Statistical analysis was conducted using the SPSS version 27 (SPSS Inc, Chicago, Illinois, USA).

## Results

Regarding the diagnosis of BD, DSA and clinical tests were in complete 100% accordance. All patients who were diagnosed as brain dead according to DSA criteria were also diagnosed as brain dead, according to the standardized clinical tests, performed by our physicians.

### CTA scoring systems compared to DSA (52 patients)

**Figure 3:**
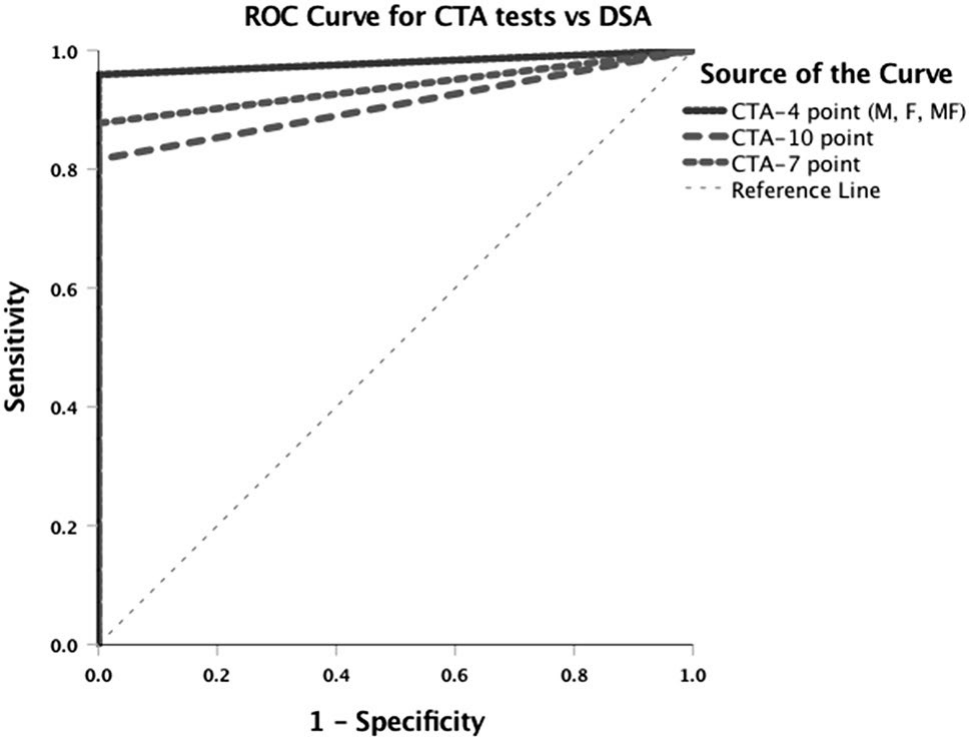
Receiver-operating characteristic curve shows the diagnostic accuracy of all computed tomography angiography (CTA) tests compared to Digital Subtraction Angiography (DSA) for determination of brain death.

**Figure 4:**
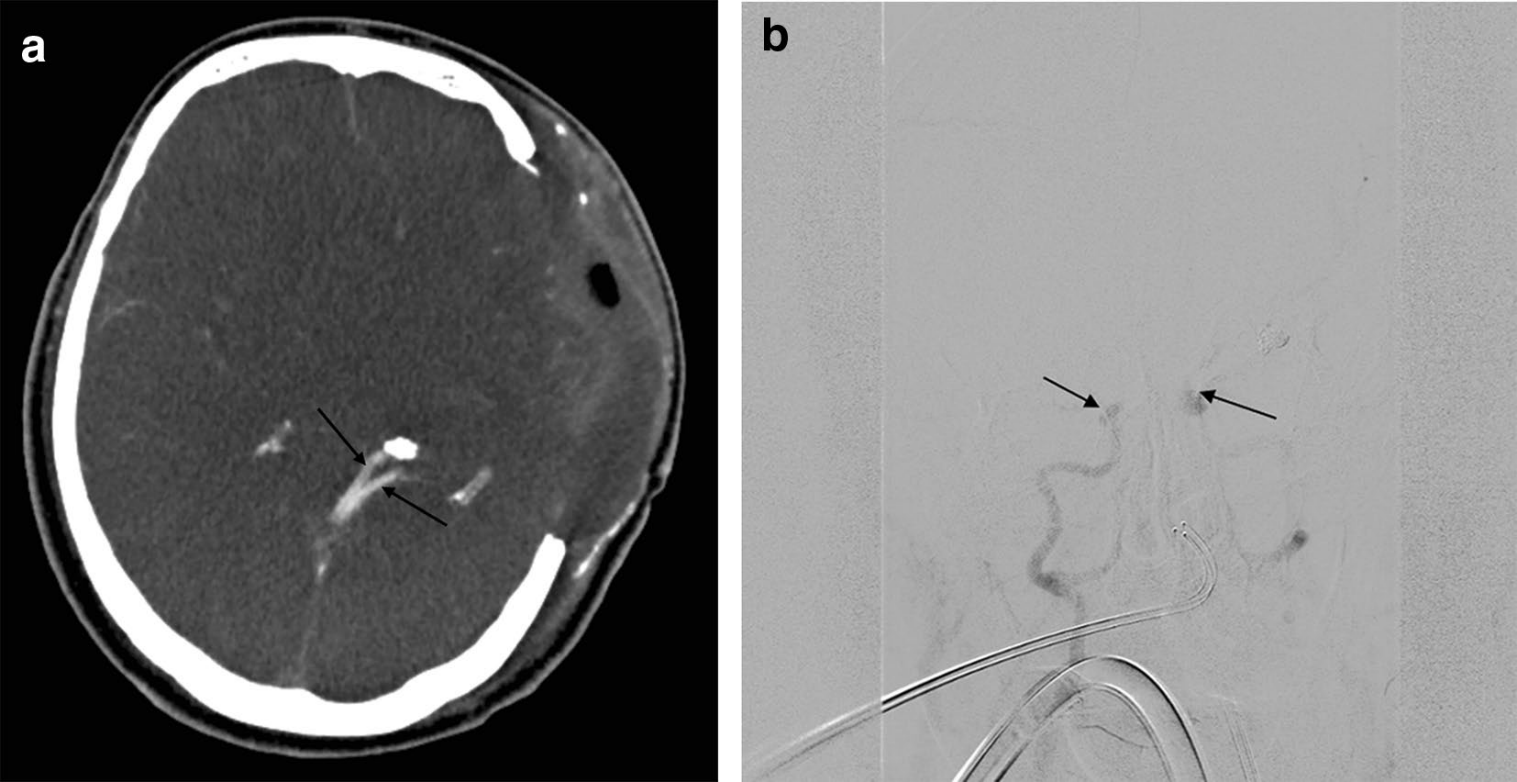
(**a**) Axial CTA (60 s phase), shows opacification of both internal cerebral veins (black arrows) in a craniectomy patient. (**b**) Digital Subtraction Angiography of the same patient in late phase (AP view), shows stasis filling on both cavernous ICAs (black arrows), with no venous outflow.

All CTA scoring systems showed 100% specificity and had a 100% PPV compared to DSA (Table 2 and Fig. 3). The various CTA scoring systems differed in their sensitivities and NPVs compared to DSA. The CTA 10-point scoring system showed the lowest sensitivity and NPV (81.6% and 25% respectively). The CTA-7-point scoring system showed 87.8% sensitivity and a 33.3% NPV compared to DSA. All CTA-4-point scoring systems (M, F and MD) showed the same sensitivity and NPV (95.9% and 60% respectively) (Table 2, Fig. 3). In 2 patients, all CTA 4-point scoring systems (M, F and MF) showed false negative results, when compared to DSA (Fig. 4a,b).

**Table 2:** Comparison of different computed tomography angiography tests (CTA) to digital subtraction angiography (DSA) in the determination of brain death.

CTA Tests							
		4 point (M, F and MF)	P-value	7 point	P-value	10 point	P-value
CTA vs DSA (52 patients)	Sensitivity	95.9 (47/49)	0.5	87.8 (43/49)	0.031	81.6 (40/49)	0.04
	Specificity	100 (3/3)		100 (3/3)		100 (3/3)	
	PPV	100 (47/47)		100 (43/43)		100 (40/40)	
	NPV	60 (3/5)		33.3 (3/9)		25 (3/12)	
	FN	4.1 (2/49)		12.2 (6/49)		9/49 (18.4)	
	AUC	0.98		0.94		0.91	
CTA vs clinical tests (49 patients)	Sensitivity	95.9 (47/49)	0.5	87.8 (43/49)	0.031	81.6 (40/49)	0.04
	PPV	100 (47/47)		100 (43/43)		100 (40/40)	
	FN	4.1 (2/49)		12.2 (6/49)		9/49 (18.4)	

Sensitivity, specificity, positive predictive value (PPV), negative predictive value (NPV) and false negative (FN) results along with area under the curve (AUC) of different CTA tests (computed tomography angiography) for determination of brain death in comparison to DSA (Digital Subtraction Angiography) and clinical tests.

Data are presented as percentages (%) and patients count for sensitivity, specificity, PPV, NPV and FN and area for AUC curve.

All comparisons among ancillary imaging tests (CTA tests and DSA or CTA and Clinical tests) for the diagnosis of brain death revealed statistical significance (p < 0.001, Cochran’s Q test).

P-value indicates differences between CTA-4 point (M, F and MF), CTA-7 point and CTA-10 point compared to DSA and clinical tests (McNemar’s test).

The sensitivities and proportions of false negative results among all ancillary tests differed significantly (p < 0.001, Cochran’s Q test, Table 2). The analysis of differences between scoring systems revealed statistically significant differences between the CTA-10 point (p = 0.004) and CTA-7 point scoring systems (p = 0.031) compared to DSA but there was no statistically significant difference between all CTA-4 point scores (M, F and MF) and DSA (p = 0.5, McNemar’s test, Table 2).

Sex, age and the presence of SDs (no, minor or major), were analysed as independent predictors of a false negative CTA results for all CTA scoring systems compared to DSA results. The presence of major SDs appeared to be an independent predictive factor of a false negative results in the CTA in CTA-4-point scoring systems (M, F and MF). For the CTA-4 point scoring systems (M, F and MF) the relative risk of false negative CTA results in patients with major SDs was 8 times higher than that in those without SDs (p = 0.008, 95% CI 1.1–58). More specifically, both patients with false negative results in the CTA-4-point scoring systems also had major SDs.

According to our results all three CTA-4 point scoring systems (M, F and MF) showed exactly the same diagnostic accuracy compared to DSA with an AUC = 98% (Fig. 3); thus, these 4-point scoring systems could be considered excellent alternatives to DSA test for BD diagnosis especially in patients with positive BD results.

### CTA compared to clinical tests (49 patients)

Clinical testing for the determination of BD was performed only in patients who were diagnosed as brain dead with DSA (49 of 52 patients, 94%). All CTA scoring systems showed a 100% PPV compared to clinical tests. The CTA-10-point scoring system showed the least sensitivity (81.6%). The CTA-7-point scoring system showed a sensitivity of 87.8% whereas all CTA-4-point (M, F and MF) scoring systems showed the highest sensitivity (95.9%) compared to clinical tests (Table 2).

The sensitivities and proportions of false negative results among all ancillary and clinical tests differed significantly (p < 0.001, Cochran’s Q test, Table 2). The analysis of differences between scoring systems revealed statistically significant differences between the CTA-10-point (p = 0.004) and CTA-7-point (p = 0.031) scoring systems compared to clinical tests but there was no statistically significant difference between all CTA-4 point scoring systems (M, F and MF) and clinical tests (p = 0.5, McNemar’s test, Table 2).

The results were exactly the same regardless of whether we compared the DSA and CTA scores or the clinical and CTA scores (McNemar’s and Cochran’s Q tests, Table 2). This result was expected, as both DSA and clinical tests revealed BD in 49 patients, while in the remaining 3 patients (without clinical tests), all CTA tests and DSA were negative for BD.

Differences between all CTA scores and clinical test results according to SDs are presented in Table 3. The sensitivities of all CTA scoring systems were reduced in patients with major SDs (62,5% for the CTA-10-point and CTA-7- point scoring system, and 75% for all 4-point scoring systems).

**Table 3:** Differences between computed tomography angiography (CTA) and clinical tests results according to patients’ skull defects.

CTA vs clinical tests (49 brain dead pts)	No skull defects (33 pts)^a^		Minor skull defects (8 pts)^b^		Major skull defects (8 pts)^c^	
	Sensitivity (%)	FN (pts no, %)	Sensitivity (%)	FN (pts no, %)	Sensitivity (%)	FN (pts no, %)
4 point (M, F and MF)	100	0/33 (0)	100	0/8 (0)	75	2/8 (25)
7 point	93.3	2/33 (6.1)	87.5	1/8 (12.5)	62.5	3/8 (37.5)
10 point	84.8	5/33 (15)	87.5	1/8 (12.5)	62.5	3/8 (37.5)

*FN* False negative results (patients that were positive for BD according to clinical tests but negative for BD according to CTA tests).

^a^ P = 0.008, ^b^ P = 0.39, ^c^ P = 0.06 (Cochran’s Q test).

In patients with or without minor SDs, all CTA 4-point scoring systems (M, F and MF) were 100% sensitive. The overall sensitivities of the CTA-10-point and CTA-7-point scoring systems compared to clinical tests in patients with no/minor SDs were 85,4% and 92,7% respectively.

## Discussion

Overall, all CTA 4-point scoring systems (CTA-F, CTA-M, and CTA-MF) proved to be more sensitive than the 10- and 7-point scoring systems. Furthermore, they were equally sensitive when compared to either DSA or clinical tests. More specifically, the CTA -10, -7 and 4-point scoring systems had overall sensitivities of 81.6%, 87.8% and 95.9% respectively. When compared to DSA or clinical tests, the differences between the CTA 7-point (p = 0.031) and CTA-10 point (p = 0.004) scoring systems were statistically significant, while all CTA 4-point scores scoring systems (M, F and MF) were not significantly different (p = 0.5). Moreover, all CTA scoring systems showed 100% specificity compared to DSA and had a 100% PPV compared to DSA and clinical tests.

**Figure 5:**
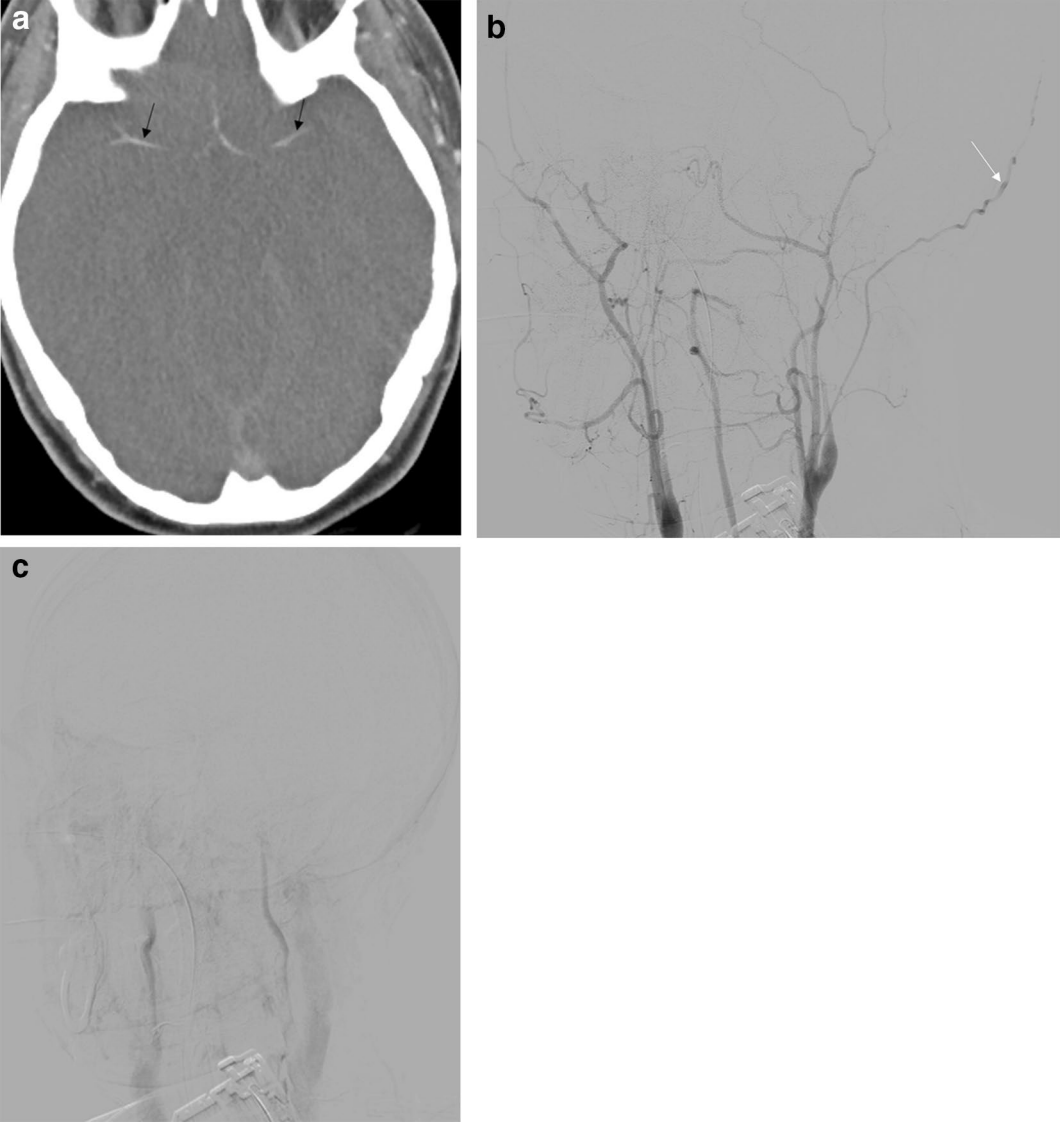
(**a**) Axial CTA (60 s phase) at the level of Circle of Willis, shows the stasis filling phenomenon, with opacification of proximal intracranial vessels (black arrows). (**b**) In the same patient, Digital Subtraction Angiography in arterial phase (AP view), shows no intracranial flow, while extracranial arteries are patent (white arrow). (**c**) In the same patient, Digital Subtraction Angiography in venous phase (AP view), shows no venous outflow.

It is well known from data published thus far that the sensitivity of CTA increases as the number of points in different scoring systems decreases. This is reflected not only in several hallmark single-centre studies, but also in meta-analyses or review papers^16,21–26^. It is also quite clear from the literature over the last 20 years, that CTA results for BD show tremendous variations in sensitivity (62–100%)^26^. These variations are due to the “misinterpretation” of SF in CTA examinations for BD. SF is a common false negative radiographic finding in CTA^11,27,28^. It is essentiallly the opacification of proximal brain vessels due to the propagation of contrast agent from the heart functioning as a pump. It does not represent an effective factor of brain parenchyma perfusion via capillaries; therefore, its presence does not preclude BD^18,29^ (Fig. 5a–c).

SF appears mainly in the proximal/intermediate intracranial arterial segments (intracranial carotid, A1-3, and M1-3 segments). Because the “arterial opacification” due to SF does not represent an effective flow for the perfusion of the brain parenchyma, the peripheral arterial flow and the venous outflow are generally not influenced by this phenomenon, especially in patients with an intact skull.

The older scoring systems (10- and 7- point scorin systems) produce many false-negative results for BD because vessels that are influenced by SF are included in the evaluation. In contrast, the more recent 4-point scoring systems (either venous or arterial) do not include these SF-dependent vessels, thereby increasing the sensitivity of CTA, by overcoming the SF effect^10,12,15,16^. In some studies the modified Frampas criteria suggested by Nunes, have been used for the same purpose^18,30^. A recent meta-analysis by Brasil et al. reported an overall sensitivity of 87.5% and showed that the 4-point scoring systems, were more sensitive than the 10- or 7-point scoring system, as in our case series.

Our goal was not only to compare all current CTA scoring systems with DSA and clinical tests, but also to evaluate the diagnostic performance of CTA scoring systems in patients with a suspicion of BD according to their workups, in subgroups with different SDs. To the best of our knowledge, this had not been evaluated in the past and is one of the strengths of our study.

It is well known that CTA results in patients with major SDs could be false negative, as extensive fractures or an operation may decrease the intracranial pressure and subsequently alter the intracranial haemodynamics, thus decreasing its diagnostic accuracy.

In a very interesting study by Nunes et al.^18^, the 4-point scoring systems were evaluated in subgroups of patients with different SD (no, minor and major) and the results showed that in patients with major SDs, CTA was less sensitive for BD confirmation (60%). Comparable results regarding CTA performance (4-point systems) in patients with open SDs were reported by Akdogan et al., in a very recent study^30^.

Our study showed similar results regarding a decrease in sensitivity; in patients with major SDs, the sensitivities of the CTA − 10-, − 7- and − 4-point scoring systems for BD, compared to clinical tests were only 62,5%, 62,5% and 75% respectively. Moreover, the presence of major SDs proved to be an independent predictive factor of false negative CTA results in the 4-point scoring systems. For the CTA-4-point scoring systems (M, F and MF), the relative risk of a false negative CTA results in patients with major SDs was 8 times higher than that in those without major SDs (p = 0.008, 95% CI 1.1–58). Finally, our study showed that the modified-Frampas score did not increase the sensitivity of CTA, in patients with major SD.

Subgroup analysis in relation to SD stratification showed that in patients with no major SDs, all 4-point scoring systems showed 100% sensitivity and 100% specificity compared to DSA and 100% sensitivity compared to clinical tests. For the same group of patients, the performance of the older 10-point and 7-point scoring systems was inferior, with a sensitivities of 85,4% and 92,7% respectively.

Furthermore, a very interesting qualitive difference was evident in patients with major SDs, although no difference was observed between the different 4-point scoring systems (CTA-F, CTA-MF and CTA-M). More specifically, the false negative results were due to the opacification of ICVs in patients who underwent craniectomy (Fig. 4a,b). In both cases, the SPVs were not opacified, but the cortical M4 segments were opacified. Although this did not changed the overall performance, the sign of non-opacified SPVs could be helpful to confirm BD in patients who underwent craniectomy.

A possible explanation for the presence of ICV opacification in patients who undergo craniectomy, is that the presence of craniectomy leads to a reduction in intracranial pressure and deep venous resistance. This, however, contrasts with the results published by Marchand et al.; at the time they introduced their revised score, they reported 100% sensitivity in patients who underwent craniectomy. Interestingly, in their study, there was no case of ICV *or* SPV opacification in patients who underwent craniectomy, and they assumed that the “intracranial arterial pressure decrease was sufficient to enhance cerebral arterial trunks but not to opacify deep veins”^16^. In our series, in both patients in whom CTA was negative for BD, the SPVs were not opacified. Therefore, in patients, who undergo craniectomy, the opacification of ICVs can be seen with CTA (even in cases of BD), and CTA negative results (using all 4-point scoring systems) should be interpreted with caution. In such cases, SPV non-opacification could be a reliable sign for BD, despite some difficulties in the assessment of SPV opacification.

Our protocol differs from all but one used in previous studies because in our clinical practice, angiographic tests precede clinical tests, and this is another strength of our study. In another similar study, Brasil et al.^19^, evaluated two ancillary tests [(CTA and transcranial Doppler (TCD)] prior to clinical tests, both in comatose patients and in patients with a clinical suspicion of BD. Moreover, DSA was not performed to verify the results of CTA and TCD. As in our study, the authors also waived clinical tests in patients with negative angiographic results for BD, while CTA showed a sensitivity between 55–96%, depending on the scoring system and the time between the ancillary and clinical tests. In their study, the venous 4-point scoring system by Marchand (CTA-M) proved to be more accurate than the 4-point scoring system suggested by Frampas (CTA-F). Our study did not verify this finding, as all 4-point scoring systems showed the same sensitivity.

The need for accurate ancillary tests has arisen because worldwide, there are still major differences in BD confirmations, due to different legislations or different application/interpretation of clinical tests, although in the past, there was an attempt to establish international guidelines for a BD diagnosis^31^. Therefore, in real life, many centres use ancillary tests independently and in combination with clinical tests. Only very recently, was a consensus document offering guidelines for BD confirmation released^3^. Even in these latest guidelines, it has been reported that ancillary tests could play a very important role in the diagnostic workup of these patients. In this confusing background, it is very important to have reliable ancillary tests (i.e., DSA or CTA) to confirm BD^32^, since its early recognition is quite important to provide closure for the family, prevent unnecessary negative medical interventions or further support and, finally, accelerate all necessary workup for organ transplantation^33^. Therefore, in our routine clinical protocol, all patients with a clinical suspicion of BD undergo an angiographic evaluation, prior to clinical tests. One drawback of this approach is that in patients with negative DSA results, for ethical reasons, sedation cannot not be stopped, and clinical tests are not performed; thus, true negative results cannot be directly proven. If clinical tests are applied in such patients, they would most likely have negative results for BD.

In our study, three patients had negative results for both DSA and CTA tests and the cessation of deep sedation was deemed unethical; thus, clinical tests were not performed. Since all three patients died 20–30 days following the angiographic tests, we assumed that they were “brain alive” at the time of the angiographic evaluation (Fig. 1f). This assumption was derived from the knowledge that in a brain dead patient, a neurohormonal catastrophic cascade, as well as haemodynamic and endocrine dysfunction, will ultimately lead to organ failure, brain liquification and subsequent cardiac arrest, in a short interval following BD^34^. Since, in the 3 patients mentioned above, true negative results for ancillary tests could not be verified, we excluded these patients from the statistical analysis of BD confirmation, thus despite the indirect evidence of non-BD, the “specificity” of CTA or DSA, when compared to clinical tests, could not be estimated. The abovementioned fact is the major limitation of our study, regarding BD confirmation.

The delay between imaging tests and the clinical examination, theoretically may reflect a change in the clinical status of the patient in this time interval, thus introducing bias. Unfortunately, this discrepancy was unavoidable, as time was needed to complete clinical tests; thus, this represents another potential limitation of our study. However, the main argument against any bias between a “hypothetically true negative” CTA result and a true positive clinical test result because of the delay (i.e., assuming that the patient died within this interval) is the fact that in all patients, DSA (the gold standard ancillary test) was performed immediately after the CTA. The discrepancies in our study were revealed between CTA and DSA and not between DSA and clinical tests; therefore, according to our opinion, a time delay would be an issue only if DSA is not performed.

Other limitations include the retrospective nature of the study, the relatively small sample size, and the fact that inter-observer reliability was not evaluated, as the reviewers of angiographic tests (both CTAs and DSAs) came to a consensus decision.

Although recent guidelines suggest that whenever ancillary tests need to be performed, DSA is the method of choice, due to its wide availability, increasing prevalence and usage of CTA, it is worth to conducting further CTA studies in order to validate or even improve its diagnostic performance for BD.

Future plans could encompass the design of a prospective study to evaluate the performance of SPV patency alone and compared to DSA and clinical tests, which might further simplify CTA criteria for BD and be reliably applied even in patients with major SDs.

In conclusion, our study verified that CTA is an excellent alternative to DSA, for the confirmation of BD, especially in patients who do not undergo craniectomy, thus allowing for quicker planning for organ donation and transplantation. In patients who do undergo craniectomy, CTA-negative results, even based on 4-point scoring systems, should be interpreted with caution and the absence of SPV opacification could be a useful marker to identify cerebral circulatory arrest.
